# Granulocytic myeloid‐derived suppressor cell population increases with the severity of alcoholic liver disease

**DOI:** 10.1111/jcmm.14109

**Published:** 2018-12-25

**Authors:** Miaomiao Gao, Ang Huang, Zijian Sun, Ying Sun, Binxia Chang, Ji‐yuan Zhang, Zheng‐sheng Zou

**Affiliations:** ^1^ Center of Non‐Infectious Liver Diseases Peking University 302 Clinical Medical School Beijing China; ^2^ Center of Non‐Infectious Liver Diseases Beijing 302 Hospital Beijing China; ^3^ Treatment and Research Center for Infectious Diseases Beijing 302 Hospital Beijing China

**Keywords:** alcoholic liver disease, arginase, myeloid‐derived suppressor cells, severity

## Abstract

Alcoholic liver disease (ALD) is a progressive liver disease that can cause a series of complications, including cirrhosis, liver failure and hepatocellular carcinoma. Granulocytic myeloid‐derived suppressor cell (gMDSC) populations have been observed to expand in various liver diseases and to inhibit innate and adaptive immunity in patients with liver disease. However, the characteristics of gMDSCs in patients with ALD have not been studied. We studied 24 healthy controls (HCs) and 107 patients with ALD and found an accumulation of gMDSCs in the peripheral blood of patients with alcoholic liver cirrhosis (ALC). Furthermore, ALC patients with a poor prognosis displayed a significant increase in peripheral gMDSCs and showed an increased capacity for arginase I production compared to HCs. In contrast, plasma arginase I levels in ALC patients were negatively correlated with total bilirubin and international normalized ratio, two key parameters of liver damage. Importantly, gMDSCs accumulated in the livers of ALC patients, and the frequency of liver gMDSCs significantly correlated with that of peripheral gMDSCs. In addition, gMDSC enrichment in vitro significantly inhibited the function of natural killer (NK) cells, perhaps preventing the NK‐induced apoptosis of hepatic stellate cells. In summary, increased peripheral and intrahepatic gMDSC populations are present in patients with ALC and may contribute to enhancing the severity of liver cirrhosis.

## 1 INTRODUCTION

With the prevention and control of hepatitis B and the country's rapid economic growth, alcoholic liver disease (ALD) now represents a greater proportion of liver diseases in China.^1^ ALD is often caused by excessive alcohol consumption; it affects over 140 million individuals^2^ and accounts for 47.9% of all liver cirrhosis‐related deaths.^3^ ALD refers to a spectrum of diseases in people with alcoholism, including alcoholic steatosis, alcoholic hepatitis (AH) and alcoholic cirrhosis (ALC). Approximately 20%‐30% of AH cases are derived from alcoholic steatosis, and approximately 15%‐20% of AH cases progress to cirrhosis.^4^ In addition to the liver toxicity of alcohol and alcohol metabolites, a growing body of evidence has focused on the harmful role of alcohol on the immune system in ALD.^2^, ^4^, ^5^, ^6^ Few studies have sought to determine the protective role of the immune response during ALD progression.

As one of the most talked about cell types, myeloid‐derived suppressor cells (MDSCs) contribute directly to the suppression of innate and adaptive immunity through the production of arginase, reactive oxygen species (ROS) and interleukin‐10 (IL‐10).^7^, ^8^, ^9^ MDSC accumulation leads to the sequestration of most of the available cysteine, which is essential for certain processes. The absence of cysteine prevents the production of proteins related to T cell activation.^10^ In addition, cytokines such as granulocyte colony stimulating factor and granulocyte‐macrophage colony stimulating factor are increased under pathological conditions, effectively stimulating the proliferation of MDSCs. Furthermore, different MDSC subsets with various phenotypic and functional features are classified as CD33^+^CD11b^high^CD14^+^HLA^−^DR^−^CD15^−^ monocytic MDSCs (mMDSCs) and CD33^+^CD11b^high^HLA^−^DR^−^CD14^−^CD15^+^ granulocytic MDSCs (gMDSCs).^11^ Accumulating evidence indicates that gMDSCs produce high levels of arginase I, IL‐10 and transforming growth factor‐β (TGF‐β) 7, 12, 13, 14 and play a vital role in regulating the immune environment. A relatively recent study showed that an increased gMDSC population inhibits T cell responses via an arginase‐dependent pathway, thus sustaining HBV replication without overt liver injury.^11^ However, whether gMDSCs can regulate the function of immunocytes in ALD patients remains unknown.

In the present study, we characterized gMDSCs in two groups of patients to determine the correlation between gMDSCs and the severity of ALD and found that the gMDSC population was increased in ALD patients and closely associated with disease progression.

## 2 MATERIALS AND METHODS

### 2.1 Patients

Blood samples were obtained from 105 individuals with ALD, including 16 AH patients and 89 ALC patients. All ALD patients were diagnosed according to existing criteria,^15^, ^16^ and individuals with concurrent HBV, HCV, autoimmune liver disease or severe systemic disease were excluded. ALC patients were further subdivided based on the Child‐Pugh score, which is used to evaluate the prognosis of patients with ALC: A (5‐6 points), B (7‐9 points) and C (10‐15 points).^17^ Twenty‐four sex‐matched healthy individuals were enrolled as controls. The study was performed in accordance with the ethical guidelines of the 1975 Declaration of Helsinki and was approved by the Ethics Committee of Beijing 302 Hospital. Written informed consent was obtained from each subject. Baseline clinical data for all patients and healthy controls (HCs) are summarized in Table 1.

**Table 1:** Clinical features of enrolled subjects

	HC	AH	ALC
Case	24	18	89
Gender (male/female)	24/0	18/0	89/0
Age (years)	29 (21, 44)	47 (35, 67)	51 (29, 83)
WBC (10^9^/L)	6.45 (4.76, 8.11)	6.74 (3.94, 10.42)	4.50 (1.54, 29.06)
NLR	1.81 (0.95, 2.45)	1.57 (0.68, 3.26)	2.24 (0.35, 9.61)
MCV (fL)	90.05 (61.10, 96.10)	93.40 (84.70, 99.50)	98.20 (74.60, 120.70)
PLT (10^9^/L)	n/a	204 (92, 478)	71 (13, 975)
PT(s)	n/a	10.9 (9.4, 12.8)	15.10 (11.00, 27.10)
PTA (%)	n/a	99.2 (72.8, 138.4)	54.80 (24.60, 110.70)
INR	n/a	0.96 (0.81, 10.60)	1.32 (0.96, 2.42)
TBIL (μmol/L)	13.1 (8.1, 23.3)	12.2 (5.9, 405.5)	38.4 (3.2, 408.6)
ALT (IU/L)	25.5 (14, 66)	165 (54, 447)	26 (6, 1027)
AST (IU/L)	23 (17, 63)	101 (23, 637)	44 (10, 1699)
ALP (IU/L)	n/a	126 (57, 562)	121 (43, 397)
GGT (IU/L)	n/a	144 (19, 2253)	59 (12, 552)
CRE (μmol/L)	n/a	70.5 (53, 102)	68 (6.2, 213)
Maddrey score	n/a	−4.67 (−11.74, 4.99)	15.56 (−4.99, 75.14)
MELD score	n/a	−3.46 (−9.58, 3.45)	3.69 (−7.59, 18.89)
Child‐Pugh (A/B/C)	n/a	n/a	16/33/40

The data were shown as median (range), except for Case, Gender and Child‐Pugh scores.

ALP, Alkaline phosphatase; ALT, alanine aminotransferases; AST, aspartate aminotransferase; CRE, Creatinine; GGT, Gamma‐glutamyl transferase; INR, International normalized ratio; MCV, Mean corpuscular volume; n/a, not applicable; NLR, Neutrophil lymphocyte ratio; PLT, Platelet; PT, Prothrombin time; PTA, Prothrombin time activity; TBIL, total bilirubin; WBC, White blood cell.

Liver diagnostic biopsies from seven AH patients, liver tissues from six ALC patients undergoing liver transplantation, and samples from four healthy liver donors were obtained for immunohistochemical analysis.

### 2.2 Flow cytometric analysis

All antibodies were purchased from Biolegend (San Diego, CA, USA), except for anti‐CD14‐APCH7, anti‐CD3‐PerCPCy5.5, anti‐CD107a‐FITC and anti‐IFN‐γ‐PECy7, which were purchased from BD Biosciences (Franklin Lakes, NJ, USA). To determine the frequencies of gMDSCs, peripheral blood mononuclear cells were first stained with live/dead‐BV510 (Life Technologies, Waltham, MA, USA) and surface antibodies and then incubated for 20 minutes at 4°C in the dark. gMDSCs were counted as CD15^+^HLA^−^DR^−^CD14^−^ cells derived from CD33^+^CD11b^+^ myeloid cells. For intracellular staining, cells were permeabilized with the Cytofix/Cytoperm Solution Kit (BD Bioscience) and stained with anti‐arginase I‐FITC (R&D, Minneapolis, MN, USA) or anti‐IFN‐γ‐PECy7; a matched isotype antibody was used as a negative control. After staining, cells were fixed in 1% paraformaldehyde and analysed using a FACS Verse flow cytometer and FlowJo software. For morphologic analysis, flow cytometric‐isolated gMDSCs were stained with a Wright‐Giemsa kit (Solarbio, Beijing, China) following the manufacturer's instructions.

### 2.3 Cell isolation

Peripheral blood mononuclear cells were isolated from freshly heparinized blood using protocols described previously by our team.^18^ gMDSCs were isolated by CD14‐negative selection, followed by CD15‐positive selection, using anti‐CD14 and anti‐CD15 antibody‐coated magnetic beads (Miltenyi Biotech, Bergisch Gladbach, Germany). Natural killer (NK) cells were isolated by negative selection using an NK isolation kit. All assays were performed according to the manufacturers’ instructions. The purity of the separated cells ranged from 95% to 98%.

### 2.4 Cell culture

Purified NK cells cocultured with or without enriched gMDSCs (at a ratio of 1:1) were stimulated with PMA (50 ng/mL) and ionomycin (1 μg/mL) (Sigma Aldrich, St. Louis, MO, USA) at 37°C for 6 hours. Anti‐CD107a antibody was added at the beginning of the stimulation period, and monensin was added 1 hour later. To further investigate the potential suppressive mechanisms by gMDSCs, 500 μmol/L Nω‐Hydroxy‐nor‐l‐arginine, diacetate salt (nor‐NOHA; Calbiochem, Darmstadt, Germany) was added in the culture system.

### 2.5 Immunohistochemistry and confocal microscopy

Paraffin‐embedded and formalin‐fixed liver tissue sections (5 μm thick) were incubated with anti‐CD15 antibody (Novus Biological, Littleton, CO, USA) and/or anti‐CD66b antibody (BD Bioscience) overnight at 4°C after endogenous peroxidase activity was blocked with 0.3% H_2_O_2_. Then, the colorimetric substrate 3‐amino‐9‐ethyl‐carbazole (red colour) was added, followed by counterstaining with haematoxylin for single staining according to previously described protocols.^19^ Images were captured at high magnification (400×) by an Olympus CX31 microscope and Olympus FV1000 confocal microscope.^20^, ^21^

### 2.6 HPLC‐MS/MS for amino acid quantification

Plasma L‐arginine levels were determined using an API 3200 QTRAP HPLC‐LC MS/MS as described elsewhere.^11^

### 2.7 Enzyme‐linked immunosorbent assay

Commercial kits were used for detecting arginase (Hycult, Uden, The Netherlands) according to the manufacturer's protocols. The detection limit of arginase I was 13 ng/mL.

### 2.8 Statistical analysis

All data were analysed using SPSS 20.0 software. The quantitative data are presented as the mean ± SD. The Kruskal‐Wallis H nonparametric test was used for multiple comparisons among various groups, and the Mann‐Whitney *U*‐test was used to compare differences between two groups. The Wilcoxon matched‐pairs signed‐rank test was used for analyses within the same group. Correlations between variables were evaluated by Spearman's rank correlation test. For all tests, a two‐sided *P* < 0.05 was considered to indicate statistical significance.

## 3 RESULTS

### 3.1 gMDSC populations were increased in ALD patients

**Figure 1:**
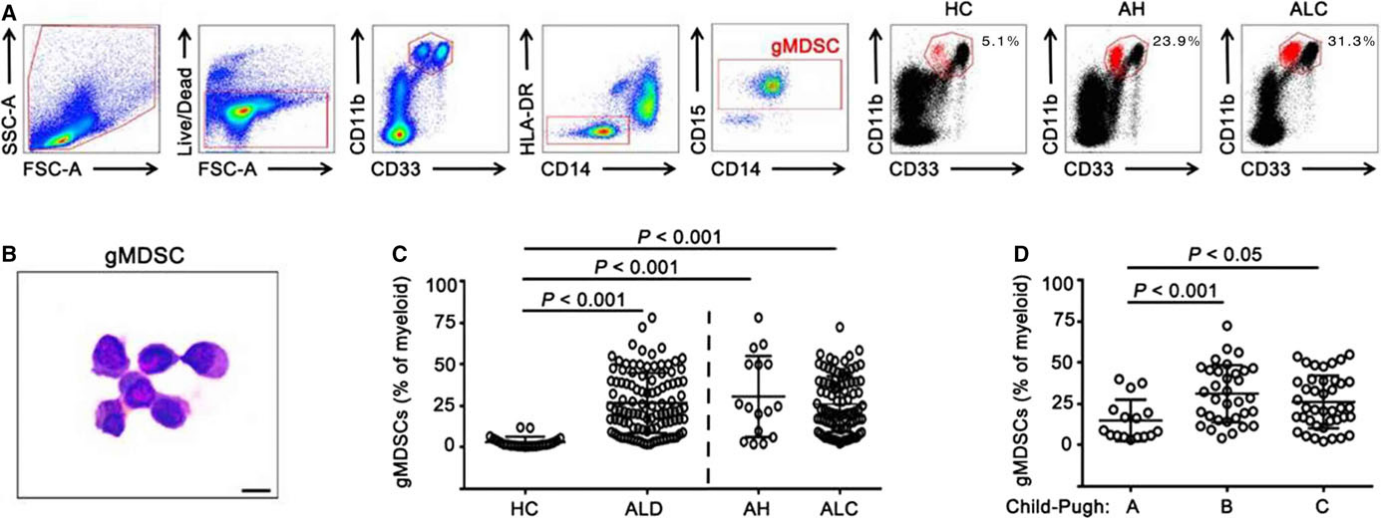
Increased frequencies of gMDSC in patients with ALD. A, Representative gating strategy for gMDSCs from freshly isolated peripheral blood mononuclear cells of healthy controls (HC), alcoholic hepatitis (AH) and alcoholic liver cirrhosis (ALC). gMDSCs were defined as the CD33^+^ CD11b^high^ HLA ^−^ DR ^−^ CD14^−^ CD15^+^ population. The gMDSC population (highlighted in red) was calculated as the proportion of myeloid cells (CD33^+^ CD11b^high^). SSC‐A, side scatter; FSC‐A, forward scatter; Live/Dead, fixable dead cell stain. B, Wright–Giemsa staining of flow cytometric‐isolated gMDSCs, bar = 10 μm. C, Cumulative data showing circulating gMDSC frequencies in 23 healthy controls and 105 ALD patients. The ALD patient group included 16 alcoholic hepatitis (AH) patients and 89 alcoholic liver cirrhosis (ALC) patients. D, Cumulative data showing circulating gMDSC frequencies analysed by Child‐Pugh score in patients with ALC. The Child‐Pugh A, B and C groups included 16, 33 and 40 patients, respectively. *P*‐values are shown

Circulating frequencies of gMDSCs were determined as the frequency of CD15^+^HLA^−^DR^−^CD14^−^ cells derived from CD33^+^CD11b^+^ myeloid cells (Figure 1A). Wright‐Giemsa staining confirmed the granulocytic nature of the gMDSCs population analysed (Figure 1B). As shown in Figure 1C, the frequency of gMDSCs was significantly higher in ALD patients than in HCs. The frequency of gMDSCs in ALD patients was highly variable; in some subjects, the expanded population of gMDSCs accounted for as much as 78% of circulating myeloid cells. Consistent with the results for gMDSCs, the frequency of mMDSCs, which were defined as the CD33^+^CD11b^high^CD14^+^HLA^−^DR^−^CD15^−^ population, was also significantly higher in ALD patients than in HCs (data not shown), but the proportion was much lower (mean = 2.48% mMDSCs vs 26.7% gMDSCs, *P* < 0.001) (Figure S1). According to the clinical diagnosis of ALD, we divided the ALD cohorts into two groups: AH and ALC. Both AH (*P* < 0.001) and ALC groups (*P* < 0.001) have a higher frequency of gMDSCs compared with HCs, whereas there was no significant difference between AH and ALC groups (*P* = 0.662) (Figure 1C).

Child‐Pugh scores are used to determine the prognosis of liver cirrhosis, and higher scores predict poor prognosis. For ALC patients, Child‐Pugh B and C patients have a greater population of gMDSCs than do Child‐Pugh A patients (Figure 1D). However, there was no significant difference between Child‐Pugh B and C patients. These data indicate that gMDSCs have a role in ALD and suggest that the increased frequency of gMDSCs in ALD patients is closely associated with disease progression.

### 3.2 Arginase^+^ gMDSCs were increased in ALD patients

**Figure 2:**
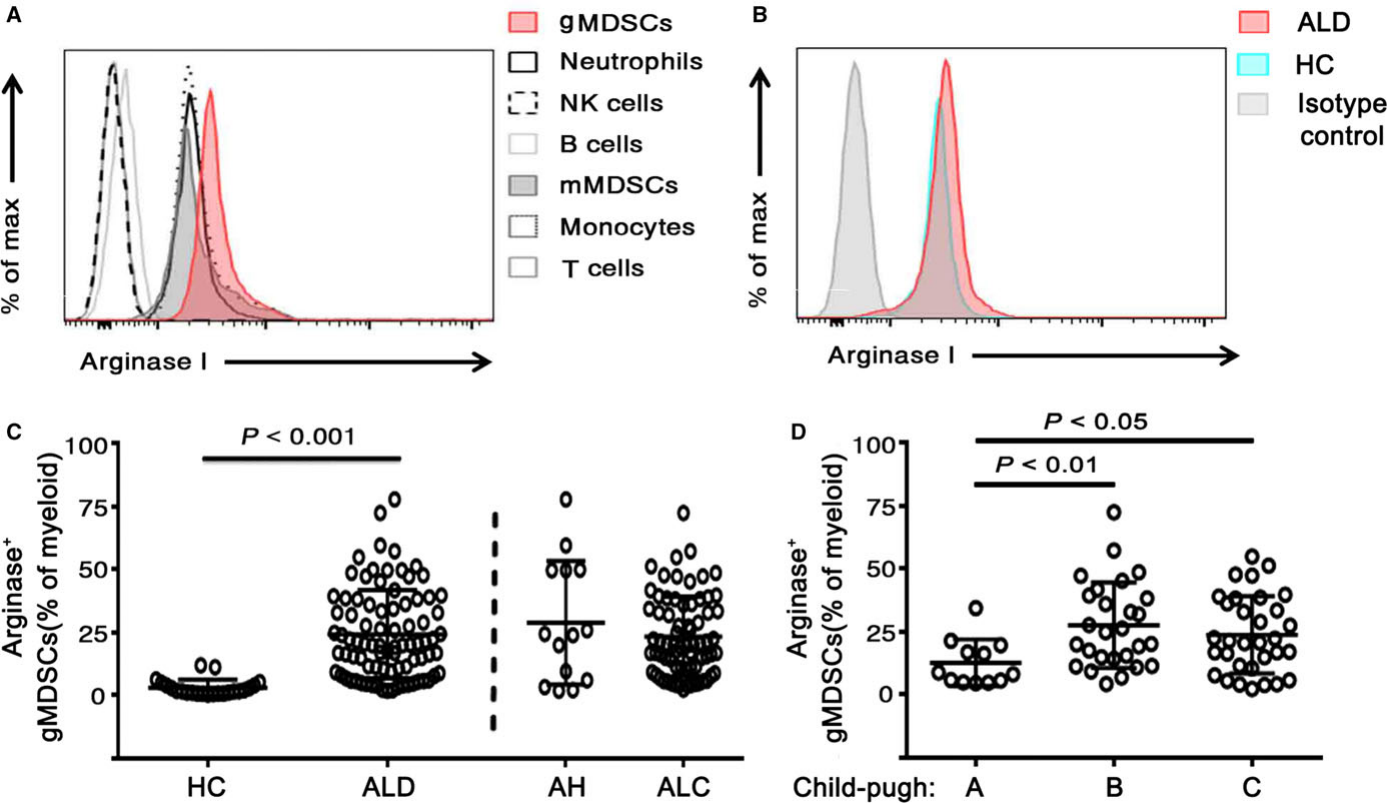
Frequencies of gMDSCs highly expressing arginase I are higher in patients with ALD. A, Analysis of intracellular arginase I by the mean fluorescence intensity (MFI) of blood cell subpopulations. Cells were identified as CD66b^+^ neutrophils, CD3^−^ CD56^+^ NK cells, CD3^−^ CD19^+^ B cells, CD33^+^ CD11b^high^ HLA ^−^ DR ^low^ CD14^+^ CD15^−^ mMDSCs, HLA ^−^ DR ^+^ CD14^+^ monocytes, and CD3^+^ T cells. B, Analysis of intracellular arginase I by the MFI in the isotype control, HC and ALD patient groups. C, Cumulative data indicating the frequencies of arginase I‐expressing gMDSCs as a percentage of myeloid cells (n = 23 healthy controls and 85 ALD patients: 14 AH and 71 ALC). D, Arginase^+^ gMDSC frequencies analyzed by Child‐Pugh score in patients with ALC (n = 12 Child‐Pugh A, 27 Child‐Pugh B, and 32 Child‐Pugh C). *P*‐values are shown

As shown in Figure 2A, gMDSCs showed stronger staining than other cell types for arginase I (Figure 2A). In the ALD and HC groups, gMDSCs showed almost 100% staining for arginase I compared to the isotype control (Figure 2B). As shown in Figure 2C, the proportion of arginase^+^ gMDSCs was elevated in both AH and ALC patients compared to HCs. Then, we analysed the difference in the arginase^+^ gMDSC frequency in ALC patients based on Child‐Pugh score. We noted a significant increase in the arginase^+^ gMDSC frequency in Child‐Pugh B and C patients compared with Child‐Pugh A patients (Figure 2D).

### 3.3 gMDSCs were enriched in the livers of ALD patients

**Figure 3:**
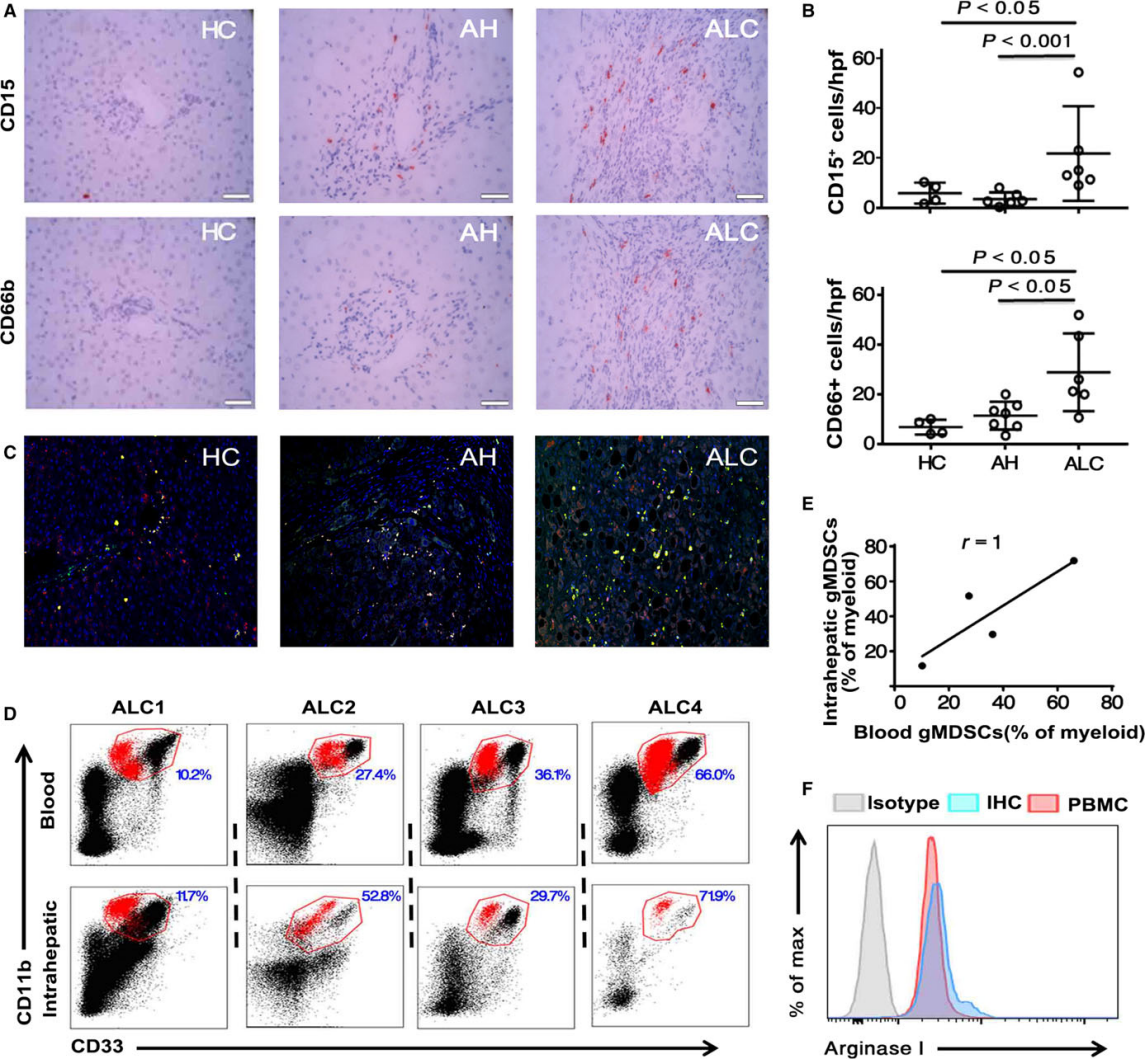
Accumulation of gMDSCs in the liver. A, Immunohistochemical staining for gMDSCs (CD15^+^ or CD66b^+^) in a portal tract of livers from a healthy control, AH patient and ALC patient (magnification, 400×). Positive cells are stained red. Scale bar, 50 μm. B, Quantitative scores for CD15^+^ or CD66b^+^ cells in the livers of four healthy controls, seven AH patients and six ALC patients (magnification, 400×). C, Representative confocal staining of the colocalization of CD15 and CD66b (in yellow) in the livers of healthy controls, AH patients and ALC patients. D, Representative dot plots of gMDSC frequencies in peripheral blood mononuclear cells (PBMCs) and livers. The percentage of gMDSCs in myeloid cells is shown in the quadrants. E, Correlation of intrahepatic gMDSC frequency with circulating gMDSC frequency. F, Analysis of intracellular arginase I by the MFI in PBMCs and livers (n = 4 ALC patients)

To determine whether gMDSCs accumulated in the liver of ALD patients, we first stained liver tissues for CD15 and CD66b, two markers of gMDSCs. As shown in Figure 3A, a few CD15‐ or CD66b‐positive cells were found in liver tissues from HCs, while the number of such cells was considerably higher in liver tissues from ALC patients than from HCs or those with AH (Figure 3B). Importantly, more CD15‐ or CD66b‐positive cells were observed in the liver sinusoids in ALC patients. These data were further validated by double epitope staining and confocal microscopy (Figure 3C). In addition, we found that ALC patients with a higher number of circulating gMDSCs had more intrahepatic gMDSCs (Figure 3D,E). In addition, both circulating and intrahepatic gMDSCs highly expressed arginase I compared with the isotype control (Figure 3F). These data suggest considerable accumulation of gMDSCs in the livers of ALC patients, and this infiltration of gMDSCs is closely associated with their peripheral counterparts.

### 3.4 Arginase I concentration was increased in ALD patients

**Figure 4:**
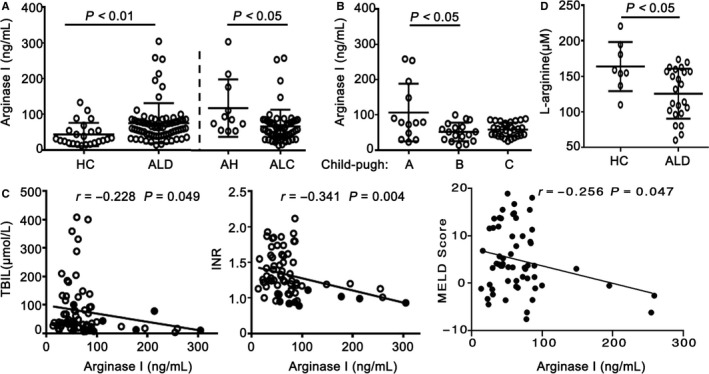
Patients with ALD have higher plasma arginase I concentrations and lower L‐arginine levels. A, Plasma concentrations of arginase I (ng/mL) were measured by ELISA (n = 24 healthy controls and 70 ALD patients: 11 AH and 59 ALC). B, Plasma arginase I concentration (ng/mL) analysed by Child‐Pugh score in patients with ALC (n = 13 Child‐Pugh A, 17 Child‐Pugh B, and 29 Child‐Pugh C). C, Correlations between plasma arginase I level and TBIL or INR (n = 70 ALD patients); the correlation between arginase I concentration and MELD score in alcoholic cirrhosis (ALC). D, Plasma L‐arginine concentration determined by HPLC‐MS/MS (n = 8 healthy controls and 23 ALD patients). *P*‐values are shown

We further analysed plasma arginase I levels and found that corresponding with the frequency of arginase^+^ gMDSCs, the plasma arginase level was markedly higher in ALD patients than in HCs. Interestingly, AH patients had a higher arginase I levels than did ALC patients (Figure 4A). In contrast with the results for arginase^+^ gMDSCs, the arginase I concentration was significantly higher in the Child‐Pugh A group than in the Child‐Pugh B group (Figure 4B). Total bilirubin (TBIL) and international normalized ratio (INR) are important indicators of the severity of liver injury. Further analysis showed that the plasma arginase I concentration was inversely correlated with TBIL and INR in ALD patients (Figure 4C). In addition, we found that the arginase I concentration was negatively with MELD score in ALC group (Figure 4C). Importantly, as shown in Figure 4D, the plasma L‐arginine concentration was significantly lower in ALD patients than in HCs.

### 3.5 gMDSCs from ALD patients showed enhanced suppressive function

**Figure 5:**
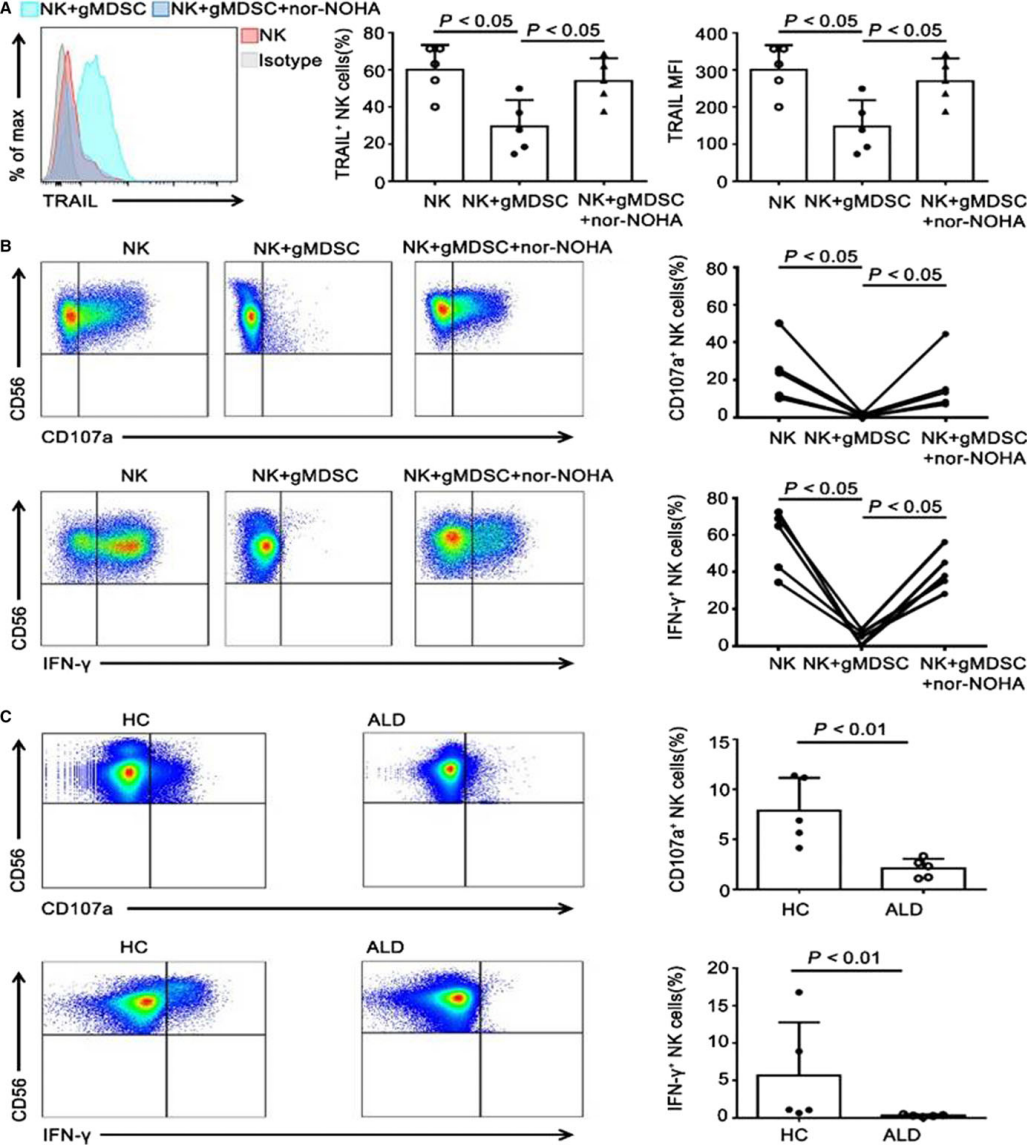
gMDSCs from ALD patients strongly suppress secretion by NK cells compared to those from HCs. A, Purified NK cells were cocultured overnight with or without enriched gMDSCs from ALD patients at a 1:5 ratio in the presence or absence of the arginase I specific inhibitor nor‐NOHA. Representative FACS histograms and cumulative TRAIL expression (percentage and MFI) on NK cells. B, Effects of arginase I specific inhibitor nor‐NOHA on gMDSCs suppression of CD107a expression and IFN‐γ production in NK cells. Representative and cumulative CD107a and IFN‐γ expression on NK cells. C, NK cells from HCs or ALD patients were cocultured for 6 h with autologous gMDSCs at a 5:1 ratio. Representative and cumulative CD107a and IFN‐γ expression on NK cells. All experimental data are from at least three independent experiments and five samples per group

Activated NK cells play an important role in attenuating liver cirrhosis by killing hepatic stellate cells (HSCs).^22^ We cultured NK cells with or without purified gMDSCs from ALD patients and found that a decreased percentage of NK cells cultured with gMDSCs expressed tumour necrosis factor‐related apoptosis‐inducing ligand (TRAIL) compared to those cultured without gMDSCs. A similar result was obtained by evaluating the TRAIL mean fluorescence intensity between the two groups (Figure 5A). Purified NK cells were stimulated with or without autologous gMDSCs. There was a lower percentage of cells expressing CD107a and a decreased capacity for IFN‐γ production among NK cells cultured with gMDSCs compared to those cultured without gMDSCs in vitro (Figure 5B). As expected, the addition of the arginase I specific inhibitor nor‐NOHA significantly restored TRAIL expression, CD107a expression, and IFN‐γ production in NK cells (Figure 5A,B). We further compared the inhibitory activity of gMDSCs from ALD patients and HCs and found that gMDSCs from ALD patients significantly inhibited CD107a expression and IFN‐γ production in NK cells (Figure 5C). These results strongly suggest that suppressive effect of gMDSCs is primarily mediated through the production of arginase in ALD patients.

## 4 DISCUSSION

Myeloid‐derived suppressor cells were first described in the early 1900s, but it was not until the late 1970s that the phenotypes, function and nomenclature of MDSCs began to be studied.^23^ In recent years, researchers have become increasingly more interested in the immunoregulatory role of MDSCs. However, until now, the characteristics of MDSCs in ALD patients had not been reported. In our study, we found that the gMDSC population was increased in ALD patients and was closely associated with disease severity. We also provide evidence for the enhanced suppressive function of gMDSCs in ALD.

Myeloid‐derived suppressor cells can be further divided into the mMDSC and gMDSC subsets based on phenotype and origin. In the hepatology field, mMDSCs play a role in chronic inflammatory liver disease. The frequencies of peripheral and intrahepatic mMDSCs correlate with liver inflammatory injury and viral load in chronic viral hepatitis^24^, ^25^ and are associated with disease severity and prognosis in primary biliary cholangitis.^26^ mMDSCs accumulate in hepatocellular carcinoma (HCC), and a higher mMDSC frequency (more than 30.5% of CD14^+^ cells) indicates a shorter survival of HCC patients treated with hepatic arterial infusion chemotherapy.^13^ There are significantly more mMDSCs in the liver in patients with non‐alcoholic fatty liver disease than in HCs.^27^ In recent years, the characteristics of gMDSCs have become an increasingly popular research topic. Expanded gMDSCs impede antiviral therapy for chronic hepatitis B patients in the immunotolerant phase.^11^ The combination of gMDSCs, PD‐1^+^ T cells and regulatory T cells (Tregs) dampens the antitumour immunity of T cells and may promote progression in patients with advanced HCC.^28^ These data suggest that gMDSCs are actively involved in liver diseases. However, little is known regarding the characteristics of gMDSCs in ALD patients. Our data in the present study revealed that increased frequencies of gMDSCs were found in patients with ALD and correlated with disease severity. There are three lines of evidence to support this finding. First, among ALC patients, those in the Child‐Pugh B and C groups had markedly elevated circulating gMDSC frequencies compared to those in the Child‐Pugh A group. Second, there was greater gMDSC accumulation in the liver of ALC patients than of HCs or AH patients. These data suggest that gMDSCs in the liver may inhibit liver inflammation. Third, the percentage of arginase^+^ gMDSCs was increased in ALC patients, and Child‐Pugh B and C patients had a significantly higher percentage of arginase^+^ gMDSCs than did Child‐Pugh A patients. Finally, in ALD patients, the arginase I concentration was increased, and plasma arginine levels were decreased. Furthermore, we observed negative correlations between arginase I concentration and TBIL or INR, both of which are key markers for assessing the prognosis of liver diseases: higher TBIL and INR values indicate a poorer prognosis. Taken together, the data indicate that gMDSCs are increased in ALD patients and associated with disease progression.

There is indisputable evidence that immune dysregulation occurs in ALD patients. In addition to neutrophils contributing to hepatic injury in ALD, other innate immune cells have been shown to play pathogenic roles in an increasing number of studies. For example, activated Kupffer cells could aggravate liver injury and fibrosis by producing ROS and tumour necrosis factor‐α via the My88 and TRIF pathways.^29^, ^30^ On the other hand, Kupffer cells ameliorate liver damage by secreting the hepatoprotective cytokine IL‐6 and the anti‐inflammatory factor IL‐10.^5^ Intrahepatic NK cells in patients with ALD could kill activated HSCs and suppress liver fibrosis.^31^ Invariant NK T cells also inhibit the number and function of NK cells by producing IL‐10.^32^ More recent studies have shown that adaptive immune cells are also involved in ALD. For example, liver CD8^+^ T cells in patients with ALD are closely associated with liver fibrosis by promoting the activation of HSCs.^4^, ^33^ In addition, elevated levels of antibodies against lipid peroxidation adducts have been observed in patients with AH.^4^

Myeloid‐derived suppressor cells have been reported to accumulate in various pathological conditions, such as cancer, inflammation, graft vs host disease and sepsis. Mechanistically, MDSCs could produce arginase and inducible nitric oxide synthase, leading to the depletion of arginine, which is essential for the T cell receptor ζ chain, resulting in impaired T cell proliferation and activation.^11^, ^19^, ^24^ mMDSCs could inhibit both HBV‐specific CD8^+^ T cell responses via PD‐1‐induced IL‐10^25^ and T cell responses through ROS‐mediated pathways during chronic HCV infection.^34^ Furthermore, gMDSCs could reduce the maturation of NK cells and drive macrophages to a suppressive M2 phenotype by secreting inhibitory cytokines such as TGF‐β and IL‐10.^9^, ^28^, ^35^ mMDSCs impair the killing ability of NK cells via NKp30, an NK cell activating receptor, and natural cytotoxicity.^36^ Lastly, MDSCs facilitate the differentiation and expansion of Tregs, which require direct cell contact.^37^, ^38^ These distinct properties of MDSCs may also occur in ALD. In the present study, we found that gMDSCs accumulated in the livers of ALC patients. Furthermore, intrahepatic gMDSCs are closely associated with their peripheral counterparts and express higher levels of arginase I. Importantly, in vitro experiments revealed that gMDSCs from ALD patients significantly suppressed IFN‐γ secretion and CD107a expression on NK cells. Moreover, gMDSCs from ALD patients had a greater ability than gMDSCs from HCs to suppress the activation and cytotoxicity of NK cells. These data suggest that increased gMDSC populations in ALD patients may aggravate liver cirrhosis by preventing the NK‐induced apoptosis of HSCs. Notably, we did not comprehensively investigate the functional characteristics of gMDSCs in ALD patients. Thus, further research is necessary to elucidate the exact function of gMDSCs in ALD patients.

In summary, this study provides the first indication that gMDSCs are increased in ALD patients and that these gMDSCs may contribute, at least in part, to the progression of ALD. This functional enhancement of gMDSCs in ALD will be of importance in the future development of gMDSC‐based therapeutic strategies for ALD.

## ACKNOWLEDGEMENTS

This study was supported by grants from the National Natural Science Foundation of China (nos. 81570538, 81670527, and 81600453), Beijing Nova Program (no. Z171100001117114) and the Military Medical Science and Technology Project of Youth Development (no. 15QNP084).

## CONFLICT OF INTEREST

The authors declare that they have no conflict of interest.
