# Supplementary figures and images for: Granulocytic myeloid‐derived suppressor cell population increases with the severity of alcoholic liver disease
Source: J Cell Mol Med. 2018 Dec 25;23(3):2032–41. doi: 10.1111/jcmm.14109 (PMC6378203; doi:10.1111/jcmm.14109)

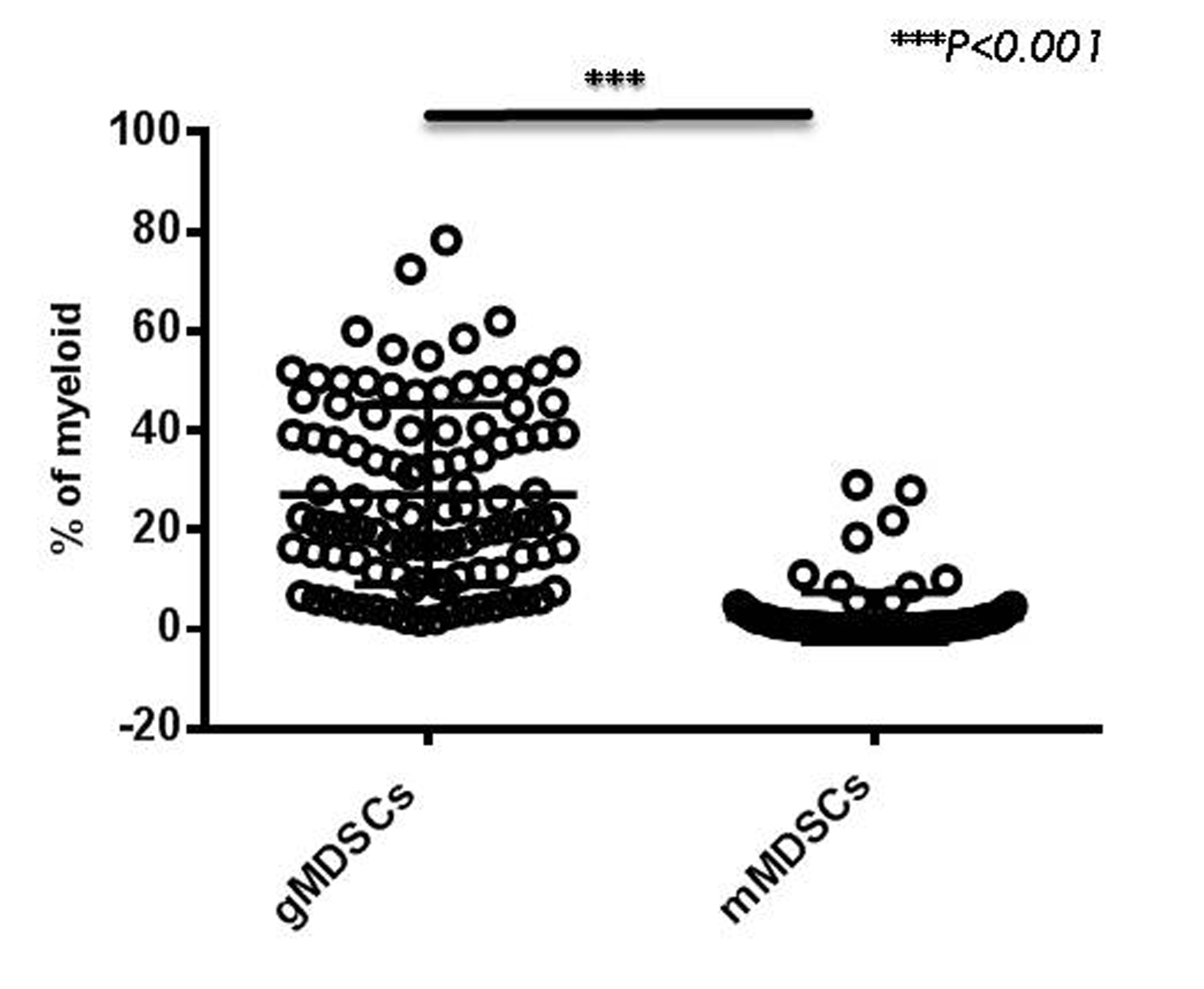

Supplement: Supplementary file 1 [file JCMM-23-2032-s001.tif]
